# Meteorological Drought Variability and Its Impact on Wheat Yields across South Africa

**DOI:** 10.3390/ijerph192416469

**Published:** 2022-12-08

**Authors:** Gift Nxumalo, Bashar Bashir, Karam Alsafadi, Hussein Bachir, Endre Harsányi, Sana Arshad, Safwan Mohammed

**Affiliations:** 1 Institute of Water and Environmental Management, Faculty of Agricultural and Food Sciences and Environmental Management, University of Debrecen, Böszörményi 138, 4032 Debrecen, Hungary; 2 Department of Civil Engineering, College of Engineering, King Saud University, P.O. Box 800, Riyadh 11421, Saudi Arabia; 3 School of Geographical Sciences, Nanjing University of Information Science and Technology, Nanjing 210044, China; 4 Department of Civil, Geo and Environmental Engineering, Technical University of Munich, 80333 Munich, Germany; 5 Institutes for Agricultural Research and Educational Farm, University of Debrecen, Böszörményi 138, 4032 Debrecen, Hungary; 6 Institute of Land Use, Technical and Precision Technology, Faculty of Agricultural and Food Sciences and Environmental Management, University of Debrecen, Böszörményi 138, 4032 Debrecen, Hungary; 7 Department of Geography, The Islamia University of Bahawalpur, Bahawalpur 63100, Pakistan

**Keywords:** water, meteorological drought, crop yield, food security, land, climate change, South Africa

## Abstract

Drought is one of the natural hazards that have negatively affected the agricultural sector worldwide. The aims of this study were to track drought characteristics (duration (DD), severity (DS), and frequency (DF)) in South Africa between 2002 and 2021 and to evaluate its impact on wheat production. Climate data were collected from the South African Weather Service (SAWS) along with wheat yield data from the Department of Agriculture, Forestry and Fisheries (2002–2021). The standard precipitation index (SPI) was calculated on 3-, 6-, 9-, and 12-month time scales, and the trend was then tracked using the Mann–Kendall (MK) test. To signify the climatic effects on crop yield, the standardized yield residual series (SYRS) was computed along with the crop-drought resilience factor (CR) on a provincial scale (2002–2021). The output of the SPI analysis for 32 stations covering all of South Africa indicates a drought tendency across the country. On a regional scale, western coastal provinces (WES-C and NR-C) have been more vulnerable to meteorological droughts over the past 20 years. Positive correlation results between SYRS and wheat yield indicate that the WES-C province was highly influenced by drought during all stages of wheat growth (Apr–Nov). Historical drought spells in 2003, 2009, and 2010 with low CR = 0.64 caused the province to be highly impacted by the negative impacts of droughts on yield loss. Overall, drought events have historically impacted the western part of the country and dominated in the coastal area. Thus, mitigation plans should be commenced, and priority should be given to this region. These findings can assist policymakers in budgeting for irrigation demand in rainfed agricultural regions.

## 1. Introduction

Climate change has been observed globally [1]. According to the Intergovernmental Panel on Climate Change (IPCC), the universal mean surface temperature is predicted to rise by 1.5–2 °C by 2100 [2,3]. The atmospheric carbon dioxide (CO_2_) concentration is expected to rise to the range of 540 to 970 parts per million around the 2100 period [4]. When the concentration levels of greenhouse gases such as carbon dioxide, methane, water vapor, and nitrogen oxide rise in the atmosphere, the Earth’s surface temperature increases [5].

This rapid increase in the Earth’s surface temperature has altered the hydrological cycle, leading to extreme weather events, such as drought, floods, heat weaves, and many others. Among them, drought has been ranked as the most damaging weather-induced disaster [6] and is considered very costly [7]. Recently, drought events have rapidly increased worldwide [8], with a catastrophic impact on ecosystems and the welfare of human beings [6]. For instance, drought killed nearly 1.3 million people between 1967 and 1991. Between 1960 and 2016, drought events killed approximately 2.2 million people and affected over 2.6 billion people worldwide. During that period (1960–2016), 669 drought events were recorded, which created USD 146 billion in economic damage [9].

Drought can be grouped into meteorological, streamflow or groundwater, and socio-economic perspectives [10]. To date, more than 100 drought indices have been suggested to analyze and characterize drought events (i.e., intensity, duration, and frequency) [8]. These indices vary in structure, computation, input, and complexity.

The most prominent of these include the standardized precipitation index (SPI) [11], reconnaissance drought index (RDI) [12], standardized precipitation evapotranspiration index (SPEI) [13], Palmer drought severity index (PDSI) [14], crop moisture index (CMI) [15], etc. The PDSI is a landmark of hydrological drought monitoring and incorporates precipitation, evaporation, runoff, and soil water recharge into the soil water balance equation. However, its complex computation and large number of input variables makes it less readily available for use [14,15,16]. The SPI is a more generalized and widely applied index for meteorological drought monitoring based on only one input, i.e., precipitation [16,17]. The performance of the SPI was further enhanced by developing the SPEI and RDI, which consider the temperature and evapotranspiration and are used for agricultural drought monitoring [18].

The World Meteorological Organization (WMO) recommended the use of the SPI in the monitoring of meteorological droughts [19]. Some other studies also used the SPI in combination with other indices to monitor droughts on global and regional scales [20,21], and it has been found to be highly correlated with the SPEI as compared to other Palmer’s indices [16]. Moreover, the versatility of the SPI to compare drought events in multiple climatic conditions at varying temporal scales makes it advantageous to use over other meteorological indices, and it is considered to be well suited for drought-related decision-making processes [22,23]. Hence, the SPI is designed to measure the rainfall deficit from the short term, i.e., SPI-1, SPI-3, and SPI-6, to the long term, i.e., SPI-12, SPI-36, and SPI-48, with negative values depicting drought and positive ones depicting moist conditions. However, the short-term SPI has been found to be well suited to address meteorological and agricultural droughts, while the long-term SPI better examines hydrological stress periods [24,25]. Thus, it has been reportedly applied to detect multiscale drought events in Eswatini [26], Cameroon [27], Hungary [8,28], Syria [19], and China [29].

Africa is prone to the adverse impacts of climatic extremes such as droughts because the continent is composed of developing economies with no proper infrastructure or adequate mitigation plans. Drought makes up the lion’s share of recurrent climate-related crises occurring across many African countries, usually with devastating consequences for agriculture, households, food, and energy security [30]. The World Meteorological Organization (WMO) report highlights that 1.3 billion African people are at risk of climatic extremes, as the continent’s average temperature warms faster than the rest of the world, even though the continent only contributes 4% of global greenhouse emissions [31]. Sub-Saharan Africa is projected to be particularly impacted by climate change since it already experiences hot weather with decreased and variable precipitation. African dryland crop and livestock farming has adapted to domestic environmental conditions, but farming income will decrease if more hot or dry spells persist [32].

Studies have been carried out to monitor meteorological and agricultural droughts at the global scale [33,34,35] and also at the regional scale across Africa [36,37]. For instance, Ayugi et al. [36] reviewed meteorological drought events across Africa, along with historical trends, impacts, and mitigation practices. Crop yield is a prominent factor in agroecology that is highly affected by meteorological droughts in drought-prone areas of Africa. Abebe et al. [38] reported that more than 50% variability in crop yield was explained by the SPI on Ethiopian agricultural land. Similar studies assessing drought affecting multiple crop yields have been conducted in Central Malawi and Ethiopia using meteorological indices such as the SPI, SPEI, and PDSI [39,40]. Other than meteorological drought indices, composite drought indices such as the crop drought vulnerability index (CDVI) derived from the SPI are also being utilized to measure the vulnerability of crop yield towards droughts [41].

South Africa is one of the drought-prone African countries and has experienced several interconnected climatic extremes, including drought [42], floods [43], and heat stress [44]. This has resulted in infrastructure destruction, agricultural damage, and the loss of human lives [45,46]. It is also the second-largest wheat-producing region in Sub-Saharan Africa, with a mix of irrigated and dryland production. However, the low relative production of wheat in this region is attributed to droughts and heat stress, along with biotic diseases [47]. The recent drought in 2015–2017 in the Western Cape (i.e., the largest wheat-producing region of South Africa) strongly affected wheat production and reduced its exports [48,49]. Thus, drought is one of the most catastrophic weather events in South Africa, with a drastic impact on the agricultural and environmental sectors [50]. Furthermore, Mpandeli et al. [51] reported a rise in the drought intensity and frequency in South Africa due to increasing temperatures and a rainfall deficit (1960–2015). In addition to this, another study conducted by Lottering et al. [52] also indicated that local areas such as “uMsinga” in the “KwaZulu-Natal” province of South Africa experienced drought-threatened agricultural productivity on small-scale farms. Thus, climatic pressure induced by droughts is largely affecting the agricultural production system in this region due to inadequate irrigation water management [53]. Future climatic models of the region also reveal an approximate 20% decline in rainfall in the next 3 decades, which will ultimately be responsible for below-average crop yields [54].

Hence, keeping in view the background of the study and region, the rainfall-based meteorological assessment of drought and its interrelationship with wheat yield is much-needed research of this time. Although some studies have been conducted in this region on a smaller scale using multiple drought indices to monitor agricultural droughts in the region [55,56], to the best of our knowledge, no specific studies have been conducted to address long-term meteorological droughts and their impact on wheat yields on a broader scale in this region. Thus, the aim of this study is two-fold, with a focus on the regional scale covering 31 meteorological stations:

1. To examine and analyze the variability in short-term drought (SPI-3) occurrence and trends at all meteorological stations in the region over a time period of the past 20 years (2002–2021);
2. To explore the impacts of short-term meteorological droughts (SPI-3) on wheat yield loss and its resistance using a standardized yield residual series (SYRS) and the crop drought resistance factor (CR) in all provinces in the region.

This study provides fruitful findings on a historical baseline to address this issue for better drought hazard management in the future at the regional level.

## 2. Materials and Methods

### 2.1. Study Area and Data Collection

**Figure 1:**
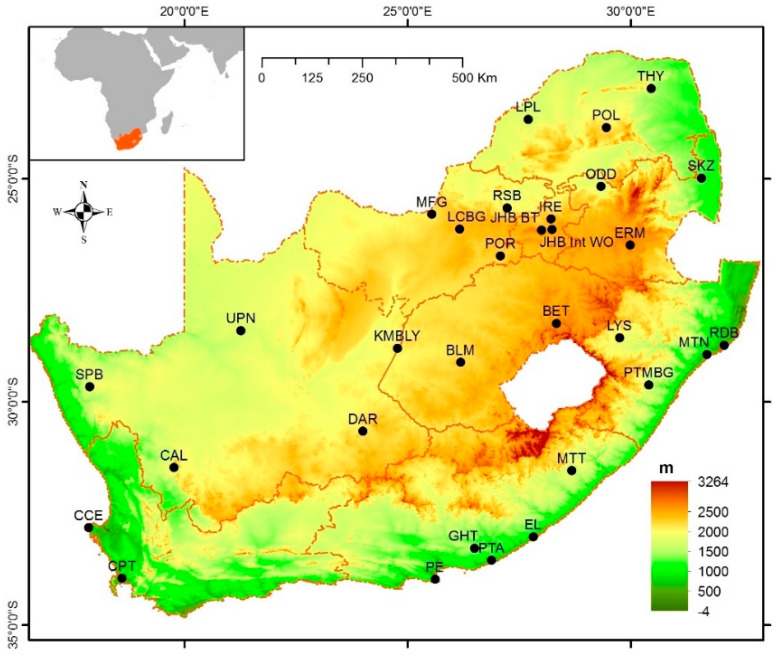
Location of South Africa, along with the distribution of the meteorological stations.

South Africa is situated between latitudes 22 and 35 °S and longitudes 17 and 33 °E and is neighbored by two oceans, the Indian Ocean in the east and the Atlantic Ocean in the west. The country spans a land area of 1,220,813 km^2^, partitions political boundaries with Mozambique, Zimbabwe, Botswana, Namibia, and Eswatini (Swaziland), and totally landlocks Lesotho [57] (Figure 1). It is described as a country with a semiarid climate that lies in the subtropics and the mid-latitudes [58].

South Africa is populated by 59.62 million people, and the major industrial activities are manufacturing, financial services, mining, tourism and trade, agriculture, and telecommunications [59]. On a regional scale, the country is divided into nine large provinces. Wheat is the main staple crop of the region after maize, with dryland and irrigated production. Climatic variations in the region make it vulnerable to extreme weather events such as drought and heat stress [47]. Rainfall in the region is quite variable from the eastern to western coastline based on the movement of oceanic currents [60]. The unpredicted rainfall variability in different seasons exposes wheat production to more climatic extremes [56].

### 2.2. Data Collection

**Figure 2:**
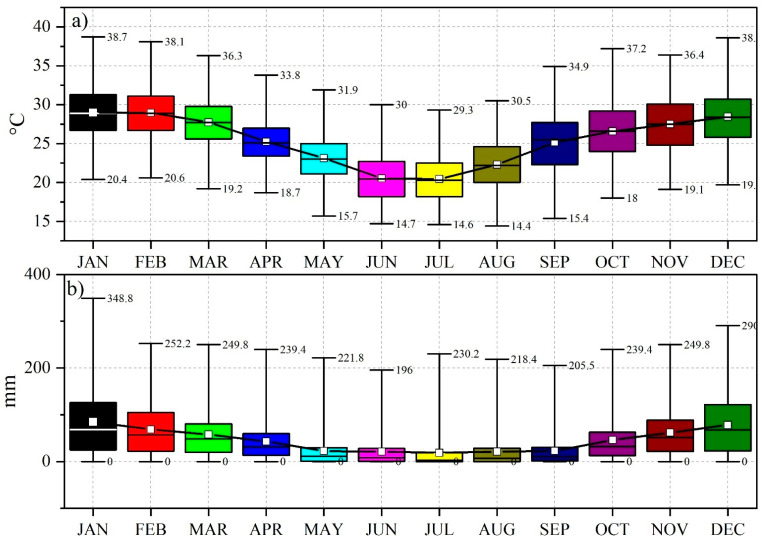
Boxplots of temperature and rainfall for all stations across South Africa (2000–2020): (**a**) temperature (°C); (**b**) rainfall (mm); (☐) mean; (^____^) median.

For the drought analysis, the available historical climatic data were commissioned from the South African Weather Service (SAWS). Rainfall and temperature data spanning the 2000–2021 period was collected from 31 meteorological stations covering the whole region of South Africa (Table 1). Data quality and homogeneity were ensured by the South African Weather Service. The average temperature was 30.22 °C in January and 23.29 °C in June, while the rainfall ranged between 108.44 mm in January and 2.5 mm in August (Figure 2).

Wheat yield data at the provincial level (Table 1) were collated from the Crop Estimates Committee of the Department of Agriculture, Forestry and Fisheries for the period from 2000 to 2021. Wheat plays a crucial role in the agricultural economy of the country [47]. The wheat growth cycle in the region prevails from April to November, with slight variations in its summer and winter characteristics [61]. Irrigated wheat is planted in summer rainfall regions in eastern states from mid-May to the end of July [62], while in winter rain areas such as WES-C, it is planted from mid-April to mid-June [63]. The harvesting period of wheat in the region runs from October to November [61].

Between 500,000 and 900,000 hectares of cereal crop is cultivated per annum, with a mean yearly output of 1.3 to 2.4 million tons between 2000 and 2021. The area under irrigation produces approximately 5 tons per hectare on an annual basis, whereas the dryland produces 2–2.5 tons per hectare [64].

### 2.3. Standard Precipitation Index (SPI)

McKee et al. [11] prescribe the standard precipitation index (SPI) to capture the spatiotemporal variation in drought properties [65]. The SPI uses long-term monthly rainfall as input data and computes the divergence in rainfall from the average numerical parameter in a certain region during a specific time span [66]. The probability density function, such as the gamma statistical distribution function, is fitted to the rainfall data, which, according to Lloyd-Hughes and Saunders in [67], fits very well. The normalization of the gamma cumulative distribution function then follows [68]. The index can assimilate the drought duration, amount, and intensity on different time scales (3, 6, 9, 12, and 24 months). More details about the SPI calculation and classification can be found in McKee et al. [11]. Table 2 shows the categories of SPI index values [11]. According to Table 2, drought can be categorized according to specific SPI values, with extreme drought corresponding to less than −2 [69,70].

### 2.4. Drought Analysis

#### 2.4.1. Drought Trend and Characteristics

The Mann–Kendall (MK) test [71] is applied to assess the monotonic tendencies of studied variables. This statistical test is nonparametric and assumes no normality but independent data [72]. In this case, the null (*H*_0_) assumes that there is no tendency in the studied variable, whereas the alternative hypothesis (*H_a_*) presumes there is a tendency [73]. In addition, the Sen slope estimator was implemented to capture the values of changes during the study period [74].

Moreover, the drought characteristics computed in this study include the drought duration (DD) in number of months and the drought sum (DS), which defines the sum of all SPI values during a particular drought spell in months or years. The frequency of drought events is calculated by employing the equation [75,76]

(1) $$\mathrm{DF}=\frac{n_{s}}{N_{s}}\times 100$$

where $n_{s}$ represents the number of drought events during the selected time, and $N_{s}$ represents the total number of months, i.e., 240 in the currently selected period of 20 years (2002–2021).

#### 2.4.2. Drought Impact on the Agricultural Sector

**Figure 3:**
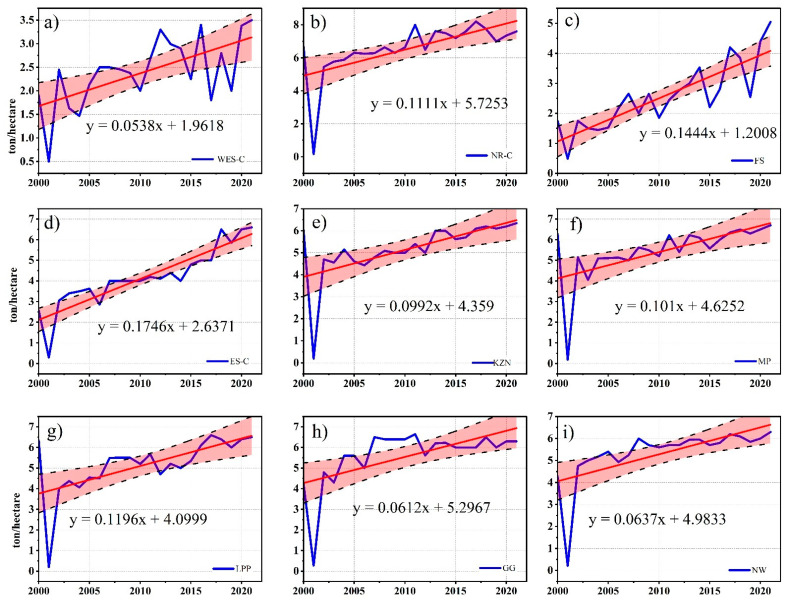
Evolution of wheat yield. Evolution of wheat yield (ton/hectare) between 2000 and 2020 in South Africa on a provincial level: (**a**) WES-C; (**b**) NR-C; (**c**) FS; (**d**) ES-C; (**e**) KZN; (**f**) MP; (**g**) LPP; (**h**) GG; (**i**) NW.

South African cereal production, particularly wheat (Figure 3), has been steadily increasing, mainly due to the expansion of cultivated land, an increase in irrigated areas, and the adoption of advanced agricultural inputs, such as improved seed cultivars [77].

Evolution of wheat yield on provincial scale over the selected time of 20 years is shown in Figure 3 where red line presents the yield trend and black dotted line presents the significance level.

To investigate the intercorrelation between agricultural drought (SPI-3) and wheat yield, the standardized yield residual series (SYRS) was calculated on a regional scale. A polynomial regression model was deployed to offset climatic, economic, and technological factors [78,79]. Yield variations due to nonclimatic factors (e.g., fertilizers, improved seed breeds, and irrigation operations) were separated using the detrending technique, and the remaining detrended yield was employed [80]. To signify the climatic effects on crop yield, the standardized yield residual series (SYRS) was computed employing the following equation:

(2) $$\mathrm{SYRS}=\frac{{\hat{X}}_{i}-\mu }{\sigma }$$

where ${\hat{X}}_{i}$ represents the residuals of the detrended yield; *μ* is the mean of the residuals of the detrended yield; and *σ* denotes the standard deviation (*i*: year). Table 3 shows the categories of the SYRS.

To showcase the impact of seasonal agrarian drought (SPI-3) on wheat production on a regional scale, the crop drought resilience factor (CR) was computed per province across South Africa. Mohammed et al. [81] define the CR as the ability of the cultivated crop to resist environmental pressures (such as drought) while maintaining its physiological, biochemical, and morphological duties. The CR [82] was calculated by the following equation:

(3) $$\mathrm{CR}=\frac{D_{dx}}{D_{\begin{array}{c} dt\\ y \end{array}}}$$

where $D_{dx}$ denotes values of yield in drought production seasons at the provincial level, and $D_{dt}$ represents values of detrended yield in a similar production season. Table 3 [82] depicts the CR categorization.

#### 2.4.3. Correlation Analysis between Crop Yields and Agricultural Drought Indices

A nonlinear regression model was employed to explore the temporal relationship between drought indices and the SYRS. This relationship was studied to determine the impact of seasonal agrarian drought (SPI-3) on wheat crop yields on a regional scale. The Pearson correlation coefficient values between the SYRS and SPI-3 were calculated to determine the provinces that were severely affected by drought in South Africa (2002–2021).

## 3. Results

### 3.1. SPI Trend and Frequency on Regional and Provincial Scales

**Figure 4:**
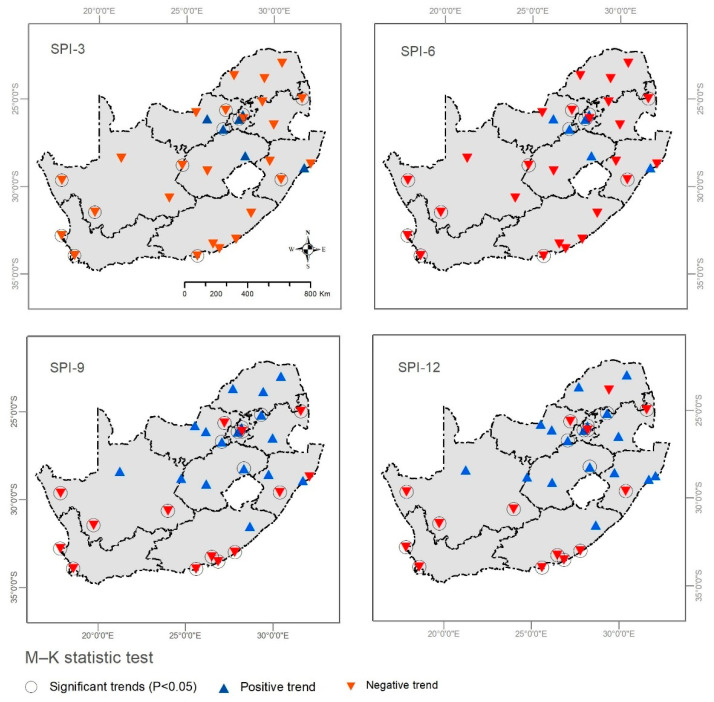
Drought (SPI) trend between 2002 and 2021 in South Africa on different time scales: 3 months; 6 months; 9 months; and 12 months.

The output of the SPI analysis reveals that the northern and eastern parts of the country were less prone to drought compared with the southern and western parts (Figure 4 and Table 4). Both SPI-3 and SPI-6 showed that the majority of the stations experienced a negative MK trend (an increase in drought events) (Table 4) and shown by inverted red triangles in Figure 4. For SPI-3, only five stations (15.6%) experienced a positive MK trend (decrease in drought events), while eight stations (28.12%) witnessed a significant (*p* < 0.05) increase in drought events (Table 4) and shown in circular inverted red triangles in Figure 4. For SPI-6, only three stations (9.3%) were less vulnerable to drought (*p* < 0.05), while the rest (90%) witnessed an increase in the drought trend. In terms of SPI-9, fourteen stations (43%) were more susceptible to drought (*p* < 0.05), while four stations depicted a significant positive (*p* < 0.05) MK trend. Like SPI-9, the MK test for SPI-12 revealed that thirteen (40.6%) stations witnessed a significant (*p* < 0.05) increase in drought events, while only two stations were significantly (*p* < 0.05) less vulnerable to drought (Table 4).

Three stations, POR, IRE, and JHB BT, exhibited a significant positive (*p* < 0.05) trend (i.e., less affected by drought) (2000–2020) on the four SPI time scales (SPI-3, 6, 9, and 12) (Figure 4 and Table 4). Interestingly, these four stations were in the northern mountains and received an average monthly rainfall of 43.94–55 mm (Table 1 and Figure 1). In contrast, nine stations, namely, RSB (northern part), SKZ (eastern part), SPB (western part), PTA (southern part), PE (southern part), PTMBG (eastern part), CAL (southern part), CCE (southern part), and CPT (southern part), showed a fixed significant negative (*p* < 0.05) trend (Figure 1 and Figure 4; Table 4). Overall, drought events historically impacted the western part of the country and dominated in the coastal area.

**Figure 5:**
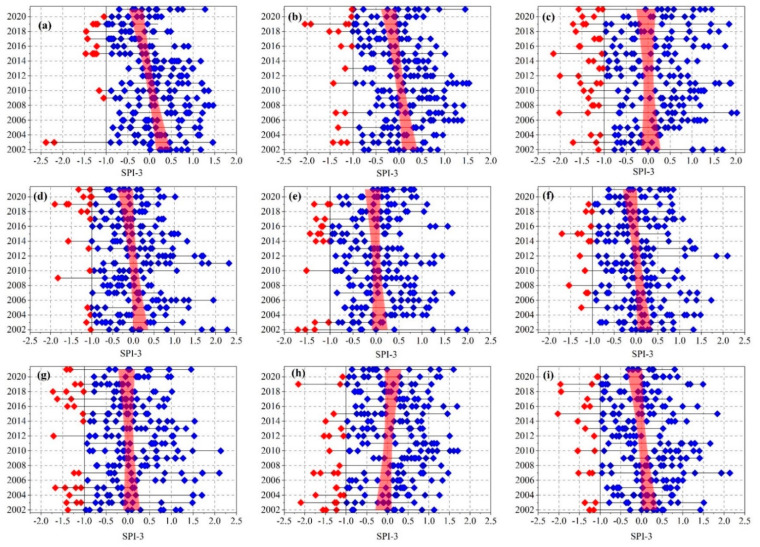
SPI-3 values on a monthly scale across South African provinces between 2002 and 2021: (**a**) WES-C; (**b**) NR-C; (**c**) FS; (**d**) ES-C; (**e**) KZN; (**f**) MP; (**g**) LPP; (**h**) GG; (**i**) NW (red dots indicate SPI-3 values of less than −1).

On a regional scale, six provinces exhibited a negative SPI-3 trend (increase in drought) between 2002 and 2021. The highest decrease was recorded in WES-C (western part of the country, *p* < 0.05), followed by NR-C and NW (Figure 5). Two provinces experienced a positive SPI-3 value, namely, LPP and GG; however, this trend was not significant (Figure 5). Looking in depth at the SPI-3 values across all the provinces, most of the provinces experienced at least one event with less than −1.5, categorized as a severe drought event. However, the lowest value (−2.4) was recorded in WES-C (Figure 5).

**Figure 6:**
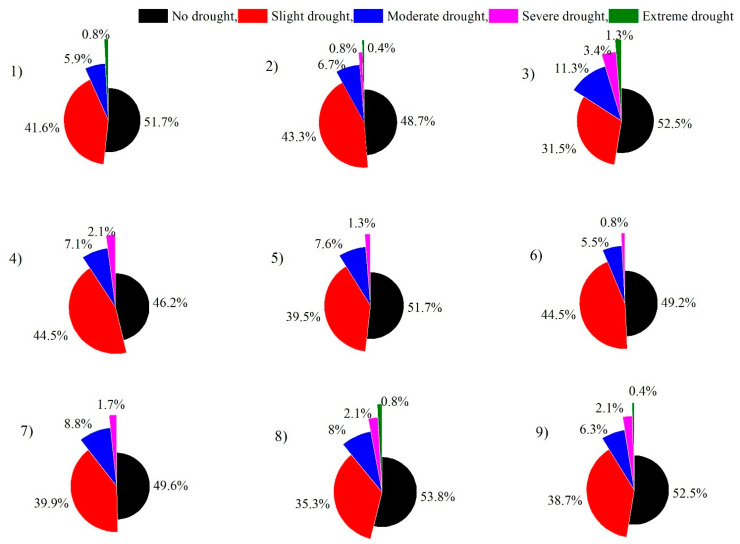
Frequency of SPI-3 drought occurrence at provincial scale between 2002 and 2021: (**1**) WES-C; (**2**) NR-C; (**3**) FS; (**4**) ES-C; (**5**) KZN; (**6**) MP; (**7**) LPP; (**8**) GG; (**9**) NW.

Figure 6 presents the percentages of drought frequencies categorized from “no drought” to “extreme drought” based on SPI-3 ranges (Table 2) in all provinces of South Africa over a period of 20 years. A total of 1.3% of months had extreme drought events in the FS province, followed by 0.8% of months in the GG province and 0.4 % in NW. Similarly, the highest percentage of moderate to severe drought months was also experienced in the FS province, i.e., 11.3 and 3.4%, followed by 8.8% of months with moderate droughts in the LPP province of the region. The WES-C province of the region experienced 41.6% of months with mild droughts, 5.9% of months with moderate droughts, and only 0.8% of months with extreme drought events over a period of 20 years. Overall, the highest percentage of drought months was experienced by the FS province in the region, which makes it vulnerable to negative impacts. Overall, the highest percentage, i.e., 53.7%, of all droughts (mild to extreme) was experienced by ES-C, followed by 51.2% in NR-C, 50.8% in MP, and 50.4% in LPP. The lowest of all drought percentages, i.e., 46.2%, was experienced in the GG province of the region.

### 3.2. Impact of Drought on Wheat Production (SYRS)

The main idea of implementing the SYRS is to isolate the impact of agricultural development on crop yield due to climate conditions. The output of the SYRS could provide an overview of the direct impact of drought on wheat production in South Africa. In this research, SPI-3 was chosen as a representative of agricultural drought, and then the SYRS was calculated on a regional scale (i.e., the nine provinces).

In the NW province, the lowest SYRS value was recorded in 2006, where SYRS*_SPI-3_* = −1.95, indicating the high impact of drought on wheat yield. In 2015, 2016, and 2019, the SYRS*_SPI-3_* values were −1.21 (moderate losses), −0.89 (acceptable losses), and −1.00 (acceptable losses), respectively. However, the impact of drought (SPI-3) in the other years could be neglected (Table 5). For the GG province in 2003, 2006, and 2012, there was a negative impact on wheat yield, where the SYRS*_SPI-3_* values were −1.89 (moderate losses), −1.59 (moderate losses), and −1.63 (moderate losses), respectively (Table 5). In the LPP province, the lowest SYRS*_SPI-3_* value was recorded in 2012 (−1.82). The drought impacts were extreme in MP, where the SYRS*_SPI-3_* value reached −2.37 in 2003. For both KZN and EsC provinces, drought had extreme impacts on wheat production, where SYRS*_SPI-3_* = −1.79 and SYRS*_SPI-3_* = −1.72, respectively. The highest value of SYRS*_SPI-3_* was recorded in 2009, revealing extreme wheat loss in the FS province. In 2012, the SYRS*_SPI-3_* reached the lowest value (−1.78) in the NR-C province (Table 5). However, the WES-C province was affected by drought in 2017 and 2019, where the SYRS*_SPI-3_* analysis showed extreme drought impacts (SYRS*_SPI-3_* = −2 (2017), SYRS*_SPI-3_* = −1.74 (2019)).

By tracking the impact of drought (SPI-3) on wheat yield across the country, three years can be distinguished based on the values of SYRS*_SPI-3_* (≤−1.5). In this sense, 2003 had a negative impact on wheat production in MP, LPP, and GG provinces. Three provinces, ES-C, KZN, and NW, were affected in 2005, while a recent drought in 2019 had a direct impact on FS, WES-C, and NR-C (Table 5).

### 3.3. Correlation between SYRS and SPI-3 on a Monthly Time Scale

The impact of drought on crop yield varied between the provinces. Table 6 depicts the correlation between SYRS and SPI-3 on a monthly scale in each province across South Africa. For WES-C, the SYRS had a positive correlation for all months. However, the highest correlations were in March (*r* _SPI-3 vs. SYRS_ = 0.53), July (*r* _SPI-3 vs. SYRS_ = 0.55), and August (*r* _SPI-3 vs. SYRS_ = 0.55) (Table 6). In NR-C, the highest correlation was recorded in the growing cycle (Table 6). Like NR-C, the highest correlation in the FS province was recorded between October (*r* _SPI-3 vs. SYRS_ = 0.54) and December (*r* _SPI-3 vs. SYRS_ = 0.54) (Table 6). For both NS-C and NKZ, there was a low correlation between SPI-3 and SYRS (Table 6). For MP, the highest correlation was recorded in May (*r* _SPI-3 vs. SYRS_ = 0.41) (Table 6). The correlation was weak in LPP from January to May and had the lowest value (*r* _SPI-3 vs. SYRS_ = 0.2, April–May) (Table 6). In the GG province, the growing cycle reflects a good correlation with SPI-3 in May (*r* _SPI-3 vs. SYRS_ = 0.35), June (*r* _SPI-3 vs. SYRS_ = 0.51), and July (*r* _SPI-3 vs. SYRS_ = 0.53) (Table 6). The growing cycle in the NW province did not reveal any notable correlation between SYRS values and drought (SPI-3) (Table 6).

### 3.4. Drought Resilience (CR) of Wheat on a Regional Scale

The CR analysis provides an overview of crop resistance to drought. In this research, the CR was calculated for the nine provinces across South Africa. All results were compared with the threshold CR = 0.8 (Table 3) to indicate whether wheat yield was moderately resilient to drought events or not. The most affected province by drought was WES-C (western part of the country), where the CR value was 0.65, followed by FS with CR = 0.65 (severely nonresilient) (Table 7). Interestingly, the SYRS analysis also revealed that WES-C had a positive correlation with SPI-3 in all months, while FS also had a positive correlation with SPI-3 in the harvesting months of Oct–Nov (Table 6), thus designating these provinces as the most affected by agricultural drought. Regarding the rest of the provinces, the CR values were above the threshold, i.e., ranging from 0.85 to 0.97, revealing a good deal of drought resilience.

Table 7 presents an extensive analysis of the yield loss percentage (YL %) in different stages of wheat growth and during different drought events. Consistent with the correlation evaluation, WES-C revealed the highest YL of 35% during the longest SPI-3 drought duration (DD) of 20 months, i.e., from August 2016 to March 2018, in the whole growing cycle (GC) of wheat, with a drought sum (DS) of 13.3. This was followed by a yield loss of 29.5% during another longer DD of 18 months from August 2018 to January 2020 in the GC of wheat with a DS of 14. WES-C also experienced a YL of 29.3% in 3 months of the sowing period (SP) in 2004 and 18.6% in 6 months from the SP to the growing period (GP) and 4 months from the GP to the harvesting period (HP) in 2003 and 2010, respectively.

Like WES-C, another province in the region i.e., FS, also experienced the highest SPI-3 drought-associated YL of 34.5% in 7 months from the growing period to the harvesting period (GP-HP) from July 2019 to Jan 2020. Other significant YLs of 20.4% and 17% in the same (GP-HP) stages of wheat growth were observed in 2010 and 2004, with short DDs of 4 and 5 months. The results also revealed GP to be most closely linked to YL, followed by HP, GC, and SP collectively in both provinces in the region (Table 7).

Similar results are found for other provinces in the region during different growth stages but with a good drought resilience of above 0.8. Another interesting finding is that a minimum of 7% to a maximum of 17.8% YL was found in NW, KZN, NR-C, and ES-C provinces of the region during different wet years when there was no drought identified (Table 7).

## 4. Discussion

### 4.1. Current and Future Drought across South Africa

In South Africa, a few studies have examined the drought trend and its intercorrelation with crop production. In this sense, this research was designed to bridge the gap regarding agricultural drought and its impact in South Africa and to highlight the region’s most vulnerable provinces to drought. Thus, local governments and policymakers can take action to minimize drought impacts and ensure food security through adaptation and mitigation plans. The output of the SPI analysis for 32 stations covering the whole of South Africa indicates a drought tendency across the country at different drought levels, e.g., SPI-3, SPI-6, SPI-9, and SPI-12 (Figure 4 and Table 4). Notably, most of the provinces showed a negative trend based on the SPI-3 analysis (Figure 4 and Figure 5). This drought trend could be explained by the El Niño–Southern Oscillation. In this sense, the recent 2016 El Niño indicated that the country is prone to drought trends [83,84]. The Pacific El Niño has a history of causing meteorological variability [85,86]. The El Niño–Southern Oscillation is reportedly causing drought periods in the northern region of the country [87]. In this context, the arid and semiarid climates of the study area, along with fluctuating rainfall, has accelerated the evolution of drought in the country [88]. However, a drought trend has been reported previously across Africa and in South Africa (Table 8).

Future climate projection by GCM models (from the 1960–2000 baseline timeframe) highlighted in the IPCC 4th Assessment Report [100] shows that South African rainfall is predicted to decrease by 2030–2060. The report mentions that the temperature is expected to increase by 1 and 3 degrees Celsius in most inland areas by 2060, while coastal areas will experience lower increases compared with the interior. This indicates that the surface wind direction and speed are predicted to change with high pressures from anticyclones in both the Pacific and Indian Oceans.

### 4.2. Drought Impacts on Wheat and Its Resilience

Since most droughts in Africa occur in the temporal and permanent domains, crop prediction models that are not based on these variable rates may give false positives. Drought has severe agricultural impacts, from the loss of income for farmers due to crop yield and livestock losses [101] to regional food security shortages [102]. Based on the SPI-3 versus SYRS analysis (Table 5 and Table 6), drought has had an impact on wheat production, especially in the western parts of the country. Pearson correlations between SYRS and SPI-3 on the monthly scale clearly show the strength of positive and negative relationships between yield loss and drought during stages of wheat growth. A positive correlation between them is found in WES-C during all stages of wheat growth, i.e., from Apr to Nov, over a period of 20 years. Severe droughts in 2003, also studied by Rouault and Richard [103], followed by low to moderate droughts in 2009 and 2010, became a cause of drought-associated yield loss here. Furthermore, after 2015, WES-C faced a continuous long-term drought condition that significantly impacted the whole wheat growing cycle and caused the highest YL of 29–35% in the whole of South Africa (Table 7) [56]. Similarly, LPP, GG, and NW provinces are also impacted by drought during different stages of wheat growth, with positive correlations between SYRS and SPI-3 from June to November, April to July, and January to April, respectively. In contrast, three provinces in the region, i.e., FS, ES-C, and KZN, revealed negative correlations between the SYRS and SPI-3 from Mar to Aug and April to December, respectively. A study by Shew et al. [47] revealed wheat yield loss in the dryland cropping system of FS due to heat stress, which is consistent with our results of significant SPI-3-associated YLs of 10.8 to 34.5% from the sowing to harvesting period (SP-HP) of wheat growth (Table 7).

Drought impacts crop risk profiling and seasonal crop-water requirements, which are fundamental to crop life. [104]. Unfortunately, only 25% of the total cropped area in the region is under irrigation [105]; therefore, the SPI-associated yield loss is quite evident in the results of our study.

A few studies have been carried out in South Africa to highlight the impact of drought on crop production. For instance, Unganai et al. [106] focused on the interaction between drought monitoring and corn yield predictions using remote sensing, with the findings highlighting the need to further investigate the severity of vegetation stress. Since most African countries do not possess long-term instrumental climate records, drought prediction using statistical methods is not prevalent. Senay and Verdin [107] allude to using modern GIS water balance algorithms to forecast seasonal drought to offset the negative impact of drought in agropastoral systems and ensure water allocation.

### 4.3. Strategies for Drought Mitigation in South Africa and Future Steps

The way to combat agricultural drought is to integrate robust sectoral strategies [108]. Adaptation strategies surely depend on the agropastoral practices that farmers are using. For example, cereal farmers may need to grow fall-sown crops and use better cultivars to offset the impact of drought. Other strategies for cereals sown in the winter include cultivars with reduced vernalization periods. Land management practices such as shifting from rainfed agriculture to irrigated agriculture can alleviate water stress in crops during drought seasons [109]. Reducing greenhouse gases by adopting precision agriculture practices will help alleviate carbon escape from most croplands [110]. Crop diversity, stubble residue management, and nutrient recycling procedures may help reduce the impact of drought [111].

Given that agricultural drought results in a persistent deficit in soil moisture content, which is associated with wilting crops [112], it is urgent for farmers and policymakers to adopt measures such as irrigation supply, water demand, and aftermath mitigation measures. The first two measures deal with water shortages, while the last addresses the socio-environmental impact of drought. Earthwork can improve water availability through the abstraction of groundwater and the construction of artificial dams, reservoirs, canals, or rivers. Non-earthwork systems can sound early warning alarms for drought detection and thus enable emergency responses, such as insurance aid, rehabilitation, and recovery measures. These types of measures strengthen institutional structures and their capacity to better prepare for drought [113].

Even though this research was based on input from 32 stations, the distribution of the climate stations covers the whole region and, thus, could be used to reach a robust result. Forthcoming studies will include engaging other climate indices, such as the SPEI and the Palmer drought severity index (PDSI), to identify the drought frequency and intensity based on different inputs. Furthermore, the ecosystem response to drought will be analyzed using satellite images.

### 4.4. Limitations of Study

Our study has some limitations that open new research questions for the future as well. Firstly, the study was limited to analyzing the meteorological drought impact on wheat yield using a single drought index, i.e., the SPI, which is comparable to other indices, such as the SPEI, PDSI, and scPDSI, for incorporating the impacts of temperature and evapotranspiration. A similar type of study for drought assessment in east Africa utilized the SPI and SPEI and presented quite similar results, with some uncertainties in the over- and underestimation of drought events [98]. However, the results of multiple indices may vary from region to region and can be utilized in the future to differentiate drought impacts in rainfed and irrigated wheat regions. In the next step, multiple indices, such as the SPEI, PDSI, and composite drought index (CDI) [114], will be used to capture drought events in South Africa and to assess their impacts on the agricultural sector.

## 5. Conclusions

Our study examined the temporal interaction of meteorological droughts and their impacts on wheat yield across the nine provinces of South Africa. A widely utilized meteorological drought index, i.e., the SPI, was used to examine the duration, frequency, and trend of meteorological droughts in dryland and irrigated provinces in the region. The SYRS and CR computed from trended and detrended wheat yields across all provinces were revealed to be significantly impacted by meteorological drought over a period of 20 years. The following major conclusions can be drawn from the results of this study:

- The frequency of drought events revealed that ES-C experienced the highest percentage of drought, i.e., 53.7%, followed by NR-C, MP, and LPP provinces of the region.
- SPI-3 trend analysis reveals a significant negative trend across many provinces in the region. Specifically, the western coastal provinces WES-C and NR-C have been more vulnerable to meteorological droughts over the past 20 years.
- Wheat yield loss analysis reveals that the highest SYRS = −2.52 was found in FS in 2019, followed by −2.37 in MP in 2003, −1.95 in NW in 2006, and −1.899 in GG and ES-C in 2003 and 2014, respectively. The most dreadful drought impacts on wheat yield were observed in the years 2015–2016, when all provinces experienced significant yield losses.
- Positive correlation results between the SYRS and wheat yield indicate that the WES-C province was highly influenced by drought during all stages of wheat growth, i.e., Apr–Nov. Historical drought spells in 2003, 2009, and 2010 and a low CR = 0.64 caused the province to be highly impacted by the negative impacts of droughts on yield loss.
- Some provinces in the region, including FS, ES-C, and KZN, were not found to be highly impacted by droughts, with negative correlations between the SYRS and SPI-3 during the wheat growth cycle from Apr to Nov.
- The WES-C and FS provinces of the region experienced the highest yield loss % during the SP-GP-HP of wheat growth stages with a CR of 0.65, indicating extremely low resilience. Overall, the growing period of wheat was found to be the most associated with yield loss, followed by the harvesting and sowing periods. Yield loss in the WES-C province is linked to the whole growing cycle in all months of wheat growth (Apr–Nov).

However, the current study efficiently examined the meteorological drought variations and related wheat yield losses in both rainfed and irrigated dryland provinces of South Africa, but still, the results of the study can be further enhanced by incorporating other indices, such as the SPEI and PDSI. Other than these, the incorporation of remote-sensing-based indices such as NDVI can also be utilized to examine drought-associated stress and yield loss. This research can be helpful in robust yield prediction modeling associated with climatic changes in the region.

Other than this, the major findings of the study also suggest adapting and focusing on drought-resistant agricultural practices in the western coastal parts of South Africa to prevent future yield loss risk. Our study recommends an immediate climate adaptation and mitigation plan to support farmers and stakeholders in combating climate change. A regional plan for climate awareness should also be formulated to protect the country’s food security.

## Figures and Tables

**Table 1:** Full overview of the analyzed meteorological stations in South Africa (2000–2021).

Station	Abbreviation	X	Y	Elevation (m)	R * (mm)	Province	Abbreviation
Betlehem	BET	−28.2496	28.3343	1688	58.25	Free State	FS
Bloemfointein	BLM	−29.1204	26.1874	1354	43		
Calvinia	CAL	−31.4819	19.7617	975	14.54	Western Cape	WES-C
Cape Columbine	CCE	−32.8278	17.8558	67	19.67		
Cape Town	CPT	−33.9631	18.6023	42	38.05		
Upington	UPN	−28.4111	21.2641	848	19.35		
De Aar	DAR	−30.6651	23.9927	1247	26.58	Northern Cape	NR-C
Kimberley	KMBLY	−28.8061	24.7698	1198	33.52		
Springbok	SPB	−29.6694	17.8788	1006	17.24		
East London	EL	−33.0357	27.8161	125	64.01	Eastern Cape	ES-C
Grahamstown	GHT	−33.2907	26.5026	642	42.11		
uMthatha	MTT	−31.5497	28.6739	742	54.68		
Port Alfred	PTA	−33.5595	26.8809	37	51.97		
Port Elizabeth	PE	−33.9864	25.6164	61	49.85		
Rusternburg	RSB	−25.6607	27.2322	1157	30.63	North-West	NW
Lichtenburg	LCBG	−26.133	26.1644	1487	48.5		
Mafikeng	MFG	−25.8037	25.5428	1279	39.57		
Ladysmith	LYS	−28.5755	29.7503	1078	52.19	KwaZulu Natal	KZN
Mntunzini	MTN	−28.9474	31.7079	65	92.8		
Pietermariesburg	PTMBG	−29.6278	30.4029	673	59.57		
Potchersroom	POR	−26.7359	27.0755	1351	43.94		
Richards Bay	RDB	−28.7378	32.0934	8	89.62		
JHB Bot Tuine	JHB BT	−26.1566	27.9991	1626	48.24	Gauteng	GG
JHB Int WO	JHB Int WO	−26.143	28.2346	1694	58.63		
Irene	IRE	−25.9105	28.2106	1523	55.31		
Polokwane	POL	−23.8576	29.4517	1228	54.29	Limpopo	LPP
Thohoyandou	THY	−22.9845	30.4583	618	59.11		
Lephalale	LPL	−23.6767	27.7051	840	31.92		
Skukuza	SKZ	−24.9926	31.588	271	42.98	Mpumalanga	MP
Oudestad	ODD	−25.18	29.33	949	33.79		
Ermelo	ERM	−26.4977	29.9838	1737	58.96		

* R (mm): average monthly rainfall for the whole period.

**Table 2:** Classification of standard precipitation index (SPI) for drought studies.

SPI Value	Category
−1.0 ≤ SPI ≤ 0	Mild event
−1.49 < SPI ≤ −1.0	Moderate event
−2.0 < SPI ≤ −1.5	Severe event
SPI ≤ −2.0	Extreme event

**Table 3:** Classification of SYRS and CR.

SYRS Values	SYRS Classes	CR Values	CR Classes
−0.5 < SYRS ≤ 0.5	Normal conditions	CR > 1	Resilient
−0.5 < SYRS ≤ −1.0	Acceptable losses due to drought	0.9 < CR < 1	Slightly nonresilient
−1.0 < SYRS ≤ −1.5	Moderate	0.8 < CR < 0.9	Moderately nonresilient
−1.5 < SYRS < −2.0	High	CR < 0.8	Severely nonresilient
SYRS ≤ −2.0	Extreme		

**Table 4:** Detailed trend of drought at the studied stations (2002–2021) based on MK test and Sen’s slope estimator.

Station	SPI-3		SPI-6		SPI-9		SPI-12	
	*p*	Sen’s *	*p*	Sen’s	*p*	Sen’s	*p*	Sen’s
BET	0.59	0.01	0.59	0.01	0	0.03	0	0.03
BLM	0.19	−0.01	0.19	−0.01	0.94	0	0.61	0
CAL	0.03	−0.02	0.03	−0.02	0	−0.03	0	−0.03
CCE	0	−0.03	0	−0.03	<0.0001	−0.04	<0.0001	−0.05
CPT	<0.0001	−0.04	<0.0001	−0.04	<0.0001	−0.05	<0.0001	−0.06
DAR	0.31	−0.01	0.31	−0.01	0.01	−0.02	0.01	−0.02
EL	0.14	−0.01	0.14	−0.01	<0.0001	−0.04	<0.0001	−0.04
ERM	0.92	0	0.92	0	0.4	0.01	0.29	0.01
GHT	0.18	−0.01	0.18	−0.01	0	−0.03	0.01	−0.03
IRE	0.01	0.02	0.01	0.02	<0.0001	0.04	< 0.0001	0.05
JHB BT	0	0.03	0	0.03	<0.0001	0.05	< 0.0001	0.05
JHB Int WO	0.08	−0.02	0.08	−0.02	0	−0.03	0	−0.03
KMBLY	0.04	−0.02	0.04	−0.02	0.35	−0.01	0.44	−0.01
LYS	0.31	−0.01	0.31	−0.01	0.94	0	0.66	0
LPL	0.85	0	0.85	0	0.71	0	0.61	0
LCBG	0.65	0	0.65	0	0.12	0.02	0.07	0.02
MFG	0.98	0	0.98	0	0.52	−0.01	0.35	−0.01
MTT	0.35	−0.01	0.35	−0.01	0.22	−0.01	0.11	−0.01
MTN	0.89	0	0.89	0	0.38	−0.01	0.3	−0.01
ODD	0.2	−0.01	0.2	−0.01	0.03	−0.01	0.04	−0.01
PTMBG	<0.0001	−0.05	<0.0001	−0.05	<0.0001	−0.08	<0.0001	−0.09
POL	0.25	−0.01	0.25	−0.01	0.12	−0.01	0.08	−0.02
PTA	0.05	−0.02	0.05	−0.02	<0.0001	−0.03	0	−0.02
PE	0.03	−0.02	0.03	−0.02	<0.0001	−0.04	<0.0001	−0.04
POR	<0.0001	0.04	<0.0001	0.04	<0.0001	0.06	<0.0001	0.06
RDB	0.47	−0.01	0.47	−0.01	0.05	−0.02	0.09	−0.01
RSB	<0.0001	−0.05	<0.0001	−0.05	<0.0001	−0.07	<0.0001	−0.08
SKZ	0.01	−0.03	0.01	−0.03	<0.0001	−0.05	<0.0001	−0.05
SPB	0	−0.04	0	−0.04	<0.0001	−0.05	<0.0001	−0.06
THY	0.82	0	0.82	0	0.31	0.01	0.09	0.02
UPN	0.07	−0.02	0.07	−0.02	0.08	−0.01	0.24	−0.01

* Sen’s: Sen’s slope estimator at decadal scale; gray shade indicates a significant (*p* < 0.05) trend.

**Table 5:** Year-by-year comparison of SYRS*_SPI-3_* changes in South African provinces from 2002 to 2021.

Year	WES-C	NR-C	FS	ES-C	KZN	MP	LPP	GG	NW
2002	0.96	0.08	0.19	−0.85	0.5	1.35	−0.55	−0.12	−0.62
2003	−0.82	0.3	−0.37	0.15	−0.33	−2.37	0.06	−1.89	0.03
2004	−1.28	0	−0.58	0.37	1.77	0.52	−1.01	0.74	0.36
2005	−0.1	0.55	−0.57	0.61	−0.68	0.24	−0.14	0.27	0.93
2006	0.49	−0.11	0.47	−1.79	−1.72	−0.02	−0.55	−1.59	−1.95
2007	0.36	−0.49	1.19	1.28	−0.55	−0.76	1.60	1.66	−0.84
2008	0.16	−0.01	−0.2	1	0.29	0.86	1.32	1.09	2.52
2009	−0.11	−1.29	0.79	0.65	−0.43	0.13	1.02	0.82	0.6
2010	−0.98	−0.77	−0.91	0.25	−0.79	−1.09	−0.01	0.59	−0.29
2011	0.26	2.41	−0.06	0.38	0.43	1.75	0.88	1	−0.11
2012	1.39	−1.78	0.27	−0.39	−1.56	−1.09	−1.82	−1.63	−0.45
2013	0.69	0.88	0.42	−0.11	2.04	1.15	−0.83	−0.3	0.5
2014	0.42	0.35	1.1	−1.89	1.62	0.47	−1.64	−0.27	0.27
2015	−0.96	−0.61	−1.67	−0.25	−0.35	−1.46	−1.05	−0.82	−1.21
2016	1.25	0.28	−0.91	−0.39	−0.47	−0.43	0.53	−0.77	−0.89
2017	−2	1.72	1.31	−1.18	0.67	0.36	1.50	−0.67	0.97
2018	−0.08	0.48	0.29	2.34	0.59	0.52	0.74	0.68	0.32
2019	−1.74	−1.49	−2.52	−0.43	−0.3	−0.37	−0.51	−0.33	−1
2020	0.96	−0.59	0.49	0.49	−0.41	−0.04	0.23	0.63	−0.31
2021	1.14	0.09	1.26	−0.23	−0.33	0.3	0.22	0.91	1.18

**Table 6:** Correlation between SYRS and SPI-3 on a monthly time scale and on a regional scale (2002–2021).

Province	JAN	FEB	MAR	APR	MAY	JUN	JUL	AUG	SEP	OCT	NOV	DEC
WES-C	0.16	0.3	0.53	0.4	0.48	0.38	0.55	0.55	0.52	0.32	0.1	−0.05
NR-C	0.54	0.32	0.06	−0.05	0.15	0.06	0.01	−0.22	−0.23	−0.13	−0.03	−0.24
FS	0.39	0.38	−0.11	−0.33	−0.6	−0.3	−0.24	0.03	0.13	0.54	0.5	0.54
ES-C	−0.17	0.02	0.14	−0.11	−0.22	−0.27	−0.04	−0.22	−0.36	−0.39	−0.23	−0.12
KZN	−0.18	0.09	−0.07	0.02	−0.14	−0.09	0.03	−0.11	−0.14	−0.2	−0.23	−0.18
MP	0.31	0.25	0.39	0.32	0.41	0.12	0.15	0.12	−0.15	0.14	−0.06	0.22
LPP	−0.05	−0.18	−0.19	−0.22	−0.2	0.12	0.53	0.59	0.25	0.04	0.25	0.05
GG	0.05	−0.11	−0.01	0.13	0.35	0.51	0.35	−0.03	−0.32	0.15	0.24	0.39
NW	0.24	0.14	0.26	0.12	0.05	−0.04	−0.05	−0.24	−0.45	−0.12	−0.12	−0.06

**Table 7:** Shows the percentage of wheat yield losses (YLs, %) caused by drought occurrences (drought event, DE) at the provincial level over a 3-month time scale during the winter wheat stages *.

**Western Cape (WES-C), CR = 0.65**						**Northern Cape (NR-C), CR = 0.93**						**Free State (FS), CR = 0.65**					
Year	YL%	DE	GS	DS	DD	Year	YL%	DE	GS	DS	DD	Year	YL%	DE	GS	DS	DD
2017	−35	2016_Aug_–18_Mar_	GC	13.3	20	2012	−8.7	-	n	n	n	2019	−34.5	2019_Jul_–20_Jan_	GP, HP	6.1	7
2019	−29.5	2018_Aug_–20_Jan_	GC	14	18	2019	−6.8	2019_Jul_–20_Mar_	GC	8.8	10	2015	−28.7	2015_Feb_–16_Apr_	SP, HP	10.5	11
2004	−29.3	2004_Mar_–4_Jul_	SP	1.4	3	2009	−6.4	2009_May_–9_Jun_	SP	0.6	2	2010	−20.4	2010_Jul_–10_Oct_	GP, HP	3.9	4
2003	−18.6	2003_Mar_–3_Aug_	SP, GP	6.6	6							2004	−17	2004_Jun_–4_Dec_	GP, HP	2.3	5
2010	−18.6	2010_Mar_–10_Oct_	GP, HP	2.9	4							2005	−15.9	2005_Ju1_–4_Sept_	GP	1.9	3
2015	−16.5	2015_Feb_–15_dec_	GC	9.4	11							2003	−10.8	2002_Nov_–4_Mar_	GC	10.8	17
**Eastern Cape (ES-C), CR = 0.97**						**KwaZulu−** **Natal (KZN), CR = 0.96**						**Mpumalanga (MP), CR = 0.92**					
Year	YL%	DE	GS	DS	DD	Year	YL%	DE	GS	DS	DD	Year	YL%	DE	GS	DS	DD
2006	−17.8	-	GP-HP	w	w	2006	−8.4	-	n	n	n	2003	−15.5	2003_Sept_–3_Dec_	GP, HP	1.9	4
2014	−14	2014_Jul_–4_Dec_	GP, HP	4.2	6	2012	−6.9	2012_May_–12_Aug_	SP, GP	2.3	4	2015	−7.5	2014_Jun_–15_Aug_	SP, GP	12	15
2017	−7.5	2016_Oct_–17_Spet_	SP, GP	6	12	2010	−3.4	2010_Jul_–10_Nov_	GP, HP	4.7	5	2010	−6	2010_Ju1_–11_Aug_	GP, HP	3.1	5
2002	−8.8	-	GP, HP	w	w							2012	−5.7	2012_Jun_–12_Aug_	SP, GP	2.8	3
2019	−2.5	2019_Oct_–20_Jan_	GP, HP	7.4	6							2007	−4.2	2007_Jan_–12_Jun_	SP	4.5	6
**Limpopo (LPP), CR = 0.79**						**Gauteng (GG), CR = 0.85**						**North−** **West (NW) CR = 0.97**					
Year	YL%	DE	GS	DS	DD	Year	YL%	DE	GS	DS	DD	Year	YL%	DE	GS	DS	DD
2012	−20.3	2012_Jan_–12_Aug_	SP, GP	6	8	2003	−14.5	2003_Sept_–4_Jan_	GC	6	5	2006	−7.2	−	n	n	n
2014	−18.3	2014_Jun_–14_Nov_	GP, HP	3.7	6	2006	−10.8	2006_May_–6_Jul_	SP, GP	2.5	3	2015	−4	2015_Jan_–15_Jun_	SP	3.8	6
2004	−17.6	2004_Jun_–05_Oct_	GC	10.6	18	2012	−10	2012_Jan_–12_Aug_	SP, GP	6.5	8	2019	−3.2	2019_Jul_–19_Nov_	GP, HP	5.8	5
2015	−14.2	2015_Mar_–15_Aug_	SP, GP	2.4	6	2015	−4.6	2015_Feb_–15_Jul_	SP, GP	2.4	6						
2005	−10.2	2004_Jun_–05_Oct_	GC	10.6	18												

* Winter wheat growing stages (GSs) include the sowing period (SP), which runs from May to June; the growing period (GP), which runs from June to September; the harvesting period (HP), which runs from September to November; and the entire growing cycle (GC), which runs from May to November. DD is drought duration, DS is drought severity, and gray shading is used to represent the YL (%) during wet events (w) or no drought event occurrences (n).

**Table 8:** Previous studies on drought trends across Africa and in South Africa.

Basin	Country	Drought Indices	Period	Output and Impact	Reference
Eastern Cape province	South Africa	Rainfall trend	1981–2018	Drought was detected in all seasons since 2015	Mahlalela et al. [86]
Karroo (Northern, Western, and Eastern Cape provinces)	South Africa	SPI	1900–2000	From 1900 to 1950, dry spell patterns were detected, whereas no visible precipitation or drought trends in 1951–2000 period	Hoffman et al. [89]
Africa	African countries	Remote sensing imagery (NDVI and HVI)	1981–2009	Impact of drought on agriculture	Rojas et al. [90]
Greater Horn of Africa	Ethiopia, Eritrea, Kenya, Rwanda, Somalia, Sudan, Uganda, Tanzania, Burundi, Djibouti, and South Sudan	SPEI	1964–2015	The previous 52 years saw rising trends in drought with different temporal and spatial patterns	Haile et al. [91]
West Africa (Volta Basin)	Benin, Togo, Mali, Ghana, Burkina Faso, and Ivory Coast	SPI	1961–2003	Frequency of droughts has increased since the 1970s	Kasei et al. [92]
Kairouan plain	Central Tunisia	SPOT VEGETATION NDVI	1998–2010	Reveals drought year in 2000–2001 and decadal persistent temporal fluctuations in agriculture	Amri et al. [93]
Hluhluwe–iMfolozi Park	KZN, South Africa	NDVI, EVI, BAI, and NDII	2002–2017	The indices show the vegetation experienced water stress, especially in 2003 and 2014–2016	Mbatha and Xulu [94]
Zambia	Africa	SPI	1981–2017	Widespread floods in DJF (summer) seasons and drought periods in 1992, 1995, and 2005	Musonda et al. [95]
Chichaoua–Mejjate	Morocco	SPI and NDVI	2008–2017	Trend in temporal persistent soaring drought with exception on 3-month SPI scale	Hadri et al. [96]
Africa	Madagascar	SPI	1900–2013	Result of drought-induced deforestation and loss of biodiversity	Desbureaux and Damania [97]
Africa	Rwanda	SPEI and SPI	1981–2020	Much variability in rainfall and temperature with a major decline in 2010–2017 and drought events in 2015, 2016, and 2017	Uwimbabazi et al. [98]
Southern Africa	Zambia	Joint UK Land Environment Simulator (JULES)	1995–2009	Strong relationships exist between drought classifications in all spatial ranges in south, west, and east regions of Zambia	Black et al. [99]

## Data Availability

Not applicable.

## References

[1] Houghton J.T., Ding Y., Griggs D.J., Noguer M., van der Linden P.J., Dai X., Maskell K., Johnson C. (2001). Climate Change 2001: The Scientific Basis: Contribution of Working Group I to the Third Assessment Report of the Intergovernmental Panel on Climate Change.

[2] Pörtner H.-O., Roberts D.C., Adams H., Adler C., Aldunce P., Ali E., Begum R.A., Betts R., Kerr R.B., Biesbroek R. (2022). Climate change 2022: Impacts, adaptation and vulnerability. IPCC Sixth Assessment Report.

[3] Zhongming Z., Linong L., Xiaona Y., Wangqiang Z., Wei L. (2021). AR6 Climate Change 2021: The Physical Science Basis.

[4] Prentice I.C., Farquhar G., Fasham M., Goulden M.L., Heimann M., Jaramillo V., Kheshgi H., Le Quéré C., Scholes R.J., Wallace D.W. (2001). The carbon cycle and atmospheric carbon dioxide. Climate Change 2001: The Scientific Basis, Intergovernmental Panel on Climate Change.

[5] Gillett N.P., Graf H.F., Osborn T.J. (2003). Climate change and the North Atlantic oscillation. Geophys. Monogr.-Am. Geophys. Union.

[6] Kogan F.N. (1997). Global drought watch from space. Bull. Am. Meteorol. Soc..

[7] Kogan F., Guo W., Yang W. (2019). Drought and food security prediction from NOAA new generation of operational satellites. Geomat. Nat. Hazards Risk.

[8] Alsafadi K., Mohammed S., Ayugi B., Sharaf M., Harsányi E. (2020). Spatial–temporal evolution of drought characteristics over Hungary between 1961 and 2010. Pure Appl. Geophys..

[9] Mekonen A.A., Berlie A.B., Ferede M.B. (2020). Spatial and temporal drought incidence analysis in the northeastern highlands of Ethiopia. Geoenviron. Disasters.

[10] Zhao L., Lyu A., Wu J., Hayes M., Tang Z., He B., Liu J., Liu M. (2014). Impact of meteorological drought on streamflow drought in Jinghe River Basin of China. Chin. Geogr. Sci..

[11] Mckee T., Doesken N., Kleist J. (1993). The relationship of drought frequency and duration to time scales. Appl. Climatol..

[12] Tsakiris G., Pangalou D., Vangelis H. (2007). Regional Drought Assessment Based on the Reconnaissance Drought Index (RDI). Water Resour. Manag..

[13] Vicente-Serrano S.M., Beguería S., López-Moreno J.I. (2010). A Multiscalar Drought Index Sensitive to Global Warming: The Standardized Precipitation Evapotranspiration Index. J. Clim..

[14] Alley W.M. (1985). The palmer drought severity index as a measure of hydrologic drought. J. Am. Water Resour. Assoc..

[15] Palmer W.C. (1968). Keeping Track of Crop Moisture Conditions, Nationwide: The New Crop Moisture Index. Weatherwise.

[16] Vicente-Serrano S.M., Beguería S., Lorenzo-Lacruz J., Camarero J.J., López-Moreno J.I., Azorin-Molina C., Revuelto J., Morán-Tejeda E., Sanchez-Lorenzo A. (2012). Performance of Drought Indices for Ecological, Agricultural, and Hydrological Applications. Earth Interact..

[17] Du J., Fang J., Xu W., Shi P. (2013). Analysis of dry/wet conditions using the standardized precipitation index and its potential usefulness for drought/flood monitoring in Hunan Province, China. Stoch. Environ. Res. Risk Assess..

[18] Xu X., Gao P., Zhu X., Guo W., Ding J., Li C. (2018). Estimating the responses of winter wheat yields to moisture variations in the past 35 years in Jiangsu Province of China. PLoS ONE.

[19] Mohammed S., Alsafadi K., Al-Awadhi T., Sherief Y., Harsanyie E., El Kenawy A.M. (2020). Space and time variability of meteorological drought in Syria. Acta Geophys..

[20] Asadi Zarch M.A., Sivakumar B., Sharma A. (2015). Droughts in a warming climate: A global assessment of Standardized precipitation index (SPI) and Reconnaissance drought index (RDI). J. Hydrol..

[21] Tirivarombo S., Osupile D., Eliasson P. (2018). Drought monitoring and analysis: Standardised Precipitation Evapotranspiration Index (SPEI) and Standardised Precipitation Index (SPI). Phys. Chem. Earth Parts A/B/C.

[22] Blain G.C. (2012). Revisiting the probabilistic definition of drought: Strengths, limitations and an agrometeorological adaptation. Bragantia.

[23] Okpara J.N., Afiesimama E.A., Anuforom A.C., Owino A., Ogunjobi K.O. (2017). The applicability of Standardized Precipitation Index: Drought characterization for early warning system and weather index insurance in West Africa. Nat. Hazards.

[24] Guttman N.B. (1999). Accepting the standardized precipitation index: A calculation algorithm 1. J. Am. Water Resour. Assoc..

[25] Yerdelen C., Abdelkader M., Eris E. (2021). Assessment of drought in SPI series using continuous wavelet analysis for Gediz Basin, Turkey. Atmos. Res..

[26] Jordaan A.J., Mlenga D.H., Mandebvu B. (2019). Monitoring droughts in Eswatini: A spatiotemporal variability analysis using the Standard Precipitation Index. Jàmbá J. Disaster Risk Stud..

[27] Guenang G.M., Kamga F.M. (2014). Computation of the standardized precipitation index (SPI) and its use to assess drought occurrences in Cameroon over recent decades. J. Appl. Meteorol. Climatol..

[28] Harsányi E., Bashir B., Alsilibe F., Alsafadi K., Alsalman A., Széles A., Rahman M.H.u., Bácskai I., Juhász C., Ratonyi T. (2021). Impact of agricultural drought on sunflower production across Hungary. Atmosphere.

[29] Jiang R., Xie J., He H., Luo J., Zhu J. (2015). Use of four drought indices for evaluating drought characteristics under climate change in Shaanxi, China: 1951–2012. Nat. Hazards.

[30] Araneda-Cabrera R.J., Bermúdez M., Puertas J. (2021). Assessment of the performance of drought indices for explaining crop yield variability at the national scale: Methodological framework and application to Mozambique. Agric. Water Manag..

[31] WMO State of the Climate in Africa. https://public.wmo.int/en/our-mandate/climate/wmo-statement-state-of-global-climate/Africa.

[32] Kurukulasuriya P., Mendelsohn R., Hassan R., Benhin J., Deressa T., Diop M., Eid H.M., Fosu K.Y., Gbetibouo G., Jain S. (2006). Will African agriculture survive climate change?. World Bank Econ. Rev..

[33] Afshar M.H., Bulut B., Duzenli E., Amjad M., Yilmaz M.T. (2022). Global spatiotemporal consistency between meteorological and soil moisture drought indices. Agric. For. Meteorol..

[34] Turco M., Jerez S., Donat M.G., Toreti A., Vicente-Serrano S.M., Doblas-Reyes F.J. (2020). A Global Probabilistic Dataset for Monitoring Meteorological Droughts. Bull. Am. Meteorol. Soc..

[35] Sánchez N., González-Zamora Á., Martínez-Fernández J., Piles M., Pablos M. (2018). Integrated remote sensing approach to global agricultural drought monitoring. Agric. For. Meteorol..

[36] Ayugi B., Eresanya E.O., Onyango A.O., Ogou F.K., Okoro E.C., Okoye C.O., Anoruo C.M., Dike V.N., Ashiru O.R., Daramola M.T. (2022). Review of meteorological drought in Africa: Historical trends, impacts, mitigation measures, and prospects. Pure Appl. Geophys..

[37] Ngcamu B.S., Chari F. (2020). Drought influences on food insecurity in Africa: A Systematic literature review. Int. J. Environ. Res. Public Health.

[38] Abebe S., Solomon A., Suryabhagavan K.V. (2021). Mapping the spatial and temporal variation of agricultural and meteorological drought using geospatial techniques, Ethiopia. Environ. Syst. Res..

[39] Chikabvumbwa S.R., Salehnia N., Manzanas R., Abdelbaki C., Zerga A. (2022). Assessing the effect of spatial–temporal droughts on dominant crop yield changes in Central Malawi. Environ. Monit. Assess..

[40] Temam D., Uddameri V., Mohammadi G., Hernandez E.A., Ekwaro-Osire S. (2019). Long-Term Drought Trends in Ethiopia with Implications for Dryland Agriculture. Water.

[41] Kamali B., Abbaspour K.C., Wehrli B., Yang H. (2018). Drought vulnerability assessment of maize in Sub-Saharan Africa: Insights from physical and social perspectives. Glob. Planet. Chang..

[42] Ziervogel G., New M., Archer van Garderen E., Midgley G., Taylor A., Hamann R., Stuart-Hill S., Myers J., Warburton M. (2014). Climate change impacts and adaptation in South Africa. Wiley Interdiscip. Rev. Clim. Chang..

[43] Olorunfemi F. (2011). Managing flood disasters under a changing climate: Lessons from Nigeria and South Africa. NISER Research Seminar Series.

[44] Nesamvuni E., Lekalakala R., Norris D., Ngambi J. (2012). Effects of climate change on dairy cattle, South Africa. Afr. J. Agric. Res..

[45] Calzadilla A., Zhu T., Rehdanz K., Tol R.S., Ringler C. (2014). Climate change and agriculture: Impacts and adaptation options in South Africa. Water Resour. Econ..

[46] Wright C.Y., Kapwata T., Du Preez D.J., Wernecke B., Garland R.M., Nkosi V., Landman W.A., Dyson L., Norval M. (2021). Major climate change-induced risks to human health in South Africa. Environ. Res..

[47] Shew A.M., Tack J.B., Nalley L.L., Chaminuka P. (2020). Yield reduction under climate warming varies among wheat cultivars in South Africa. Nat. Commun..

[48] Otto F.E., Wolski P., Lehner F., Tebaldi C., Van Oldenborgh G.J., Hogesteeger S., Singh R., Holden P., Fučkar N.S., Odoulami R.C. (2018). Anthropogenic influence on the drivers of the Western Cape drought 2015–2017. Environ. Res. Lett..

[49] Cullis J., Alton T., Arndt C., Cartwright A., Chang A., Gabriel S., Gebretsadik Y., Hartley F., de Jager G., Makrelov K. (2015). An Uncertainty Approach to Modelling Climate Change Risk in South Africa.

[50] Meza I., Rezaei E.E., Siebert S., Ghazaryan G., Nouri H., Dubovyk O., Gerdener H., Herbert C., Kusche J., Popat E. (2021). Drought risk for agricultural systems in South Africa: Drivers, spatial patterns, and implications for drought risk management. Sci. Total Environ..

[51] Mpandeli S., Nhamo L., Moeletsi M., Masupha T., Magidi J., Tshikolomo K., Liphadzi S., Naidoo D., Mabhaudhi T. (2019). Assessing climate change and adaptive capacity at local scale using observed and remotely sensed data. Weather. Clim. Extrem..

[52] Lottering S.J., Mafongoya P., Lottering R. (2021). The impacts of drought and the adaptive strategies of small-scale farmers in uMsinga, KwaZulu-Natal, South Africa. J. Asian Afr. Stud..

[53] Dube E., Tsilo T.J., Sosibo N.Z., Fanadzo M. (2020). Irrigation wheat production constraints and opportunities in South Africa. South Afr. J. Sci..

[54] Conway D., van Garderen E.A., Deryng D., Dorling S., Krueger T., Landman W., Lankford B., Lebek K., Osborn T., Ringler C. (2015). Climate and southern Africa’s water–energy–food nexus. Nat. Clim. Chang..

[55] Kganvago M., Mukhawana M.B., Mashalane M., Mgabisa A., Moloele S. Recent Trends of Drought Using Remotely Sensed and In-Situ Indices: Towards an Integrated Drought Monitoring System for South Africa. Proceedings of the 2021 IEEE International Geoscience and Remote Sensing Symposium IGARSS.

[56] Theron S., Archer E., Midgley S., Walker S. (2021). Agricultural perspectives on the 2015–2018 western cape drought, South Africa: Characteristics and spatial variability in the core wheat growing regions. Agric. For. Meteorol..

[57] Knight J., Rogerson C.M. (2019). The Geography of South Africa.

[58] Engelbrecht C.J., Landman W.A., Engelbrecht F.A., Malherbe J. (2015). A synoptic decomposition of rainfall over the Cape south coast of South Africa. Clim. Dyn..

[59] SAG South Africa at a Glance. https://www.gov.za/about-sa/south-africa-glance#:~:text=Mining%2C%20transport%2C%20energy%2C%20manufacturing%2C%20tourism%20and%20agriculture.

[60] McBride C.M., Kruger A.C., Dyson L. (2022). Changes in extreme daily rainfall characteristics in South Africa: 1921–2020. Weather. Clim. Extrem..

[61] Franch B., Cintas J., Becker-Reshef I., Sanchez-Torres M.J., Roger J., Skakun S., Sobrino J.A., Van Tricht K., Degerickx J., Gilliams S. (2022). Global crop calendars of maize and wheat in the framework of the WorldCereal project. GISci. Remote Sens..

[62] Tadesse W., Bishaw Z., Assefa S. (2018). Wheat production and breeding in Sub-Saharan Africa: Challenges and opportunities in the face of climate change. Int. J. Clim. Chang. Strateg. Manag..

[63] RSA (2016). Production Guideline for Wheat.

[64] Crop Estimate Committee. https://www.sagis.org.za/cec_reports.html.

[65] Laddimath R.S., Patil N.S., Rao P., Nagendra N. (2022). Assessing the impacts of climate change on drought-prone regions in Bhima sub-basin (India) using the Standard Precipitation Index. J. Water Clim. Chang..

[66] Zuo D.-D., Hou W., Zhang Q., Yan P.-C. (2022). Sensitivity analysis of standardized precipitation index to climate state selection in China. Adv. Clim. Chang. Res..

[67] Lloyd-Hughes B., Saunders M.A. (2002). A drought climatology for Europe. Int. J. Climatol. A J. R. Meteorol. Soc..

[68] Mathier L., Perreault L., Bobée B., Ashkar F. (1992). The use of geometric and gamma-related distributions for frequency analysis of water deficit. Stoch. Hydrol. Hydraul..

[69] Svoboda M., Hayes M., Wood D. (2012). Standardized Precipitation Index: User Guide.

[70] Kim D.-W., Byun H.-R., Choi K.-S. (2009). Evaluation, modification, and application of the Effective Drought Index to 200-Year drought climatology of Seoul, Korea. J. Hydrol..

[71] Mann H.B. (1945). Nonparametric tests against trend. Econom. J. Econom. Soc..

[72] Wasserstein R.L., Schirm A.L., Lazar N.A. (2019). Moving to a world beyond “p < 0.05”. Am. Stat..

[73] Koudahe K., Koffi D., Kayode J., Awokola S., Adebola A. (2018). Impact of climate variability on crop yields in southern Togo. Environ. Pollut. Clim. Chang..

[74] Li Z., Ali Z., Cui T., Qamar S., Ismail M., Nazeer A., Faisal M. (2022). A comparative analysis of pre- and post-industrial spatiotemporal drought trends and patterns of Tibet Plateau using Sen slope estimator and steady-state probabilities of Markov Chain. Nat. Hazards.

[75] Zambreski Z.T., Lin X., Aiken R.M., Kluitenberg G.J., Pielke Sr R.A. (2018). Identification of hydroclimate subregions for seasonal drought monitoring in the U.S. Great Plains. J. Hydrol..

[76] Gebremichael H.B., Raba G.A., Beketie K.T., Feyisa G.L. (2022). Temporal and spatial characteristics of drought, future changes and possible drivers over Upper Awash Basin, Ethiopia, using SPI and SPEI. Environ. Dev. Sustain..

[77] Nhamo L., Matchaya G., Mabhaudhi T., Nhlengethwa S., Nhemachena C., Mpandeli S. (2019). Cereal production trends under climate change: Impacts and adaptation strategies in southern Africa. Agriculture.

[78] Liu W., Kogan F. (1996). Monitoring regional drought using the vegetation condition index. Int. J. Remote Sens..

[79] Mavromatis T. (2007). Drought index evaluation for assessing future wheat production in Greece. Int. J. Climatol. A J. R. Meteorol. Soc..

[80] Wu H., Hubbard K.G., Wilhite D.A. (2004). An agricultural drought risk-assessment model for corn and soybeans. Int. J. Climatol. A J. R. Meteorol. Soc..

[81] Mohammed S., Alsafadi K., Enaruvbe G.O., Bashir B., Elbeltagi A., Széles A., Alsalman A., Harsanyi E. (2022). Assessing the impacts of agricultural drought (SPI/SPEI) on maize and wheat yields across Hungary. Sci. Rep..

[82] Sharma A., Goyal M.K. (2018). Assessment of ecosystem resilience to hydroclimatic disturbances in India. Glob. Chang. Biol..

[83] Baudoin M.-A., Vogel C., Nortje K., Naik M. (2017). Living with drought in South Africa: Lessons learnt from the recent El Niño drought period. Int. J. Disaster Risk Reduct..

[84] Nicholson S.E., Kim J. (1997). The relationship of the El Niño–Southern oscillation to African rainfall. Int. J. Climatol. A J. R. Meteorol. Soc..

[85] Chikoore H., Jury M.R. (2010). Intraseasonal variability of satellite-derived rainfall and vegetation over Southern Africa. Earth Interact..

[86] Mahlalela P., Blamey R., Hart N., Reason C. (2020). Drought in the Eastern Cape region of South Africa and trends in rainfall characteristics. Clim. Dyn..

[87] Lakhraj-Govender R., Grab S.W. (2019). Assessing the impact of El Niño–Southern Oscillation on South African temperatures during austral summer. Int. J. Climatol..

[88] Botai C.M., Botai J.O., Adeola A.M. (2018). Spatial distribution of temporal precipitation contrasts in South Africa. S. Afr. J. Sci..

[89] Hoffman M.T., Carrick P., Gillson L., West A. (2009). Drought, climate change and vegetation response in the succulent karoo, South Africa. S. Afr. J. Sci..

[90] Rojas O., Vrieling A., Rembold F. (2011). Assessing drought probability for agricultural areas in Africa with coarse resolution remote sensing imagery. Remote Sens. Environ..

[91] Haile G.G., Tang Q., Leng G., Jia G., Wang J., Cai D., Sun S., Baniya B., Zhang Q. (2020). Long-term spatiotemporal variation of drought patterns over the Greater Horn of Africa. Sci. Total Environ..

[92] Kasei R., Diekkrüger B., Leemhuis C. (2010). Drought frequency in the Volta basin of West Africa. Sustain. Sci..

[93] Amri R., Zribi M., Lili-Chabaane Z., Duchemin B., Gruhier C., Chehbouni A. (2011). Analysis of vegetation behavior in a North African semi-arid region, using SPOT-VEGETATION NDVI data. Remote Sens..

[94] Mbatha N., Xulu S. (2018). Time Series Analysis of MODIS-Derived NDVI for the Hluhluwe-Imfolozi Park, South Africa: Impact of Recent Intense Drought. Climate.

[95] Musonda B., Jing Y., Iyakaremye V., Ojara M. (2020). Analysis of long-term variations of drought characteristics using standardized precipitation index over Zambia. Atmosphere.

[96] Hadri A., Saidi M.E.M., Boudhar A. (2021). Multiscale drought monitoring and comparison using remote sensing in a Mediterranean arid region: A case study from west-central Morocco. Arab. J. Geosci..

[97] Desbureaux S., Damania R. (2018). Rain, forests and farmers: Evidence of drought induced deforestation in Madagascar and its consequences for biodiversity conservation. Biol. Conserv..

[98] Uwimbabazi J., Jing Y., Iyakaremye V., Ullah I., Ayugi B. (2022). Observed Changes in Meteorological Drought Events during 1981–2020 over Rwanda, East Africa. Sustainability.

[99] Black E., Tarnavsky E., Maidment R., Greatrex H., Mookerjee A., Quaife T., Brown M. (2016). The use of remotely sensed rainfall for managing drought risk: A case study of weather index insurance in Zambia. Remote Sens..

[100] Reddy S.S. (2000). IPCC Special Report Emission Scenarios: Summary for Plicy Makers: A Speical Report of Working Group III of Intergovernmental Panel of Climatic Change.

[101] Ding Y., Hayes M.J., Widhalm M. (2011). Measuring economic impacts of drought: A review and discussion. Disaster Prev. Manag. Int. J..

[102] Ziolkowska J.R. (2016). Socio-Economic Implications of Drought in the Agricultural Sector and the State Economy. Economies.

[103] Rouault M., Richard Y. (2005). Intensity and spatial extent of droughts in southern Africa. Geophys. Res. Lett..

[104] Rulinda C.M., Dilo A., Bijker W., Stein A. (2012). Characterising and quantifying vegetative drought in East Africa using fuzzy modelling and NDVI data. J. Arid. Environ..

[105] USDA Wheat Production in South Africa. https://ipad.fas.usda.gov/highlights/2015/09/RSA/index.htm.

[106] Unganai L.S., Kogan F.N. (1998). Drought monitoring and corn yield estimation in Southern Africa from AVHRR data. Remote Sens. Environ..

[107] Senay G.B., Verdin J. (2003). Characterization of yield reduction in Ethiopia using a GIS-based crop water balance model. Can. J. Remote Sens..

[108] Rosenzweig C., Tubiello F.N. (2007). Adaptation and mitigation strategies in agriculture: An analysis of potential synergies. Mitig. Adapt. Strateg. Glob. Chang..

[109] Rosenzweig C., Karoly D., Vicarelli M., Neofotis P., Wu Q., Casassa G., Menzel A., Root T.L., Estrella N., Seguin B. (2008). Attributing physical and biological impacts to anthropogenic climate change. Nature.

[110] Zhang W.-f., Dou Z.-x., He P., Ju X.-T., Powlson D., Chadwick D., Norse D., Lu Y.-L., Zhang Y., Wu L. (2013). New technologies reduce greenhouse gas emissions from nitrogenous fertilizer in China. Proc. Natl. Acad. Sci. USA.

[111] Holland J.M. (2004). The environmental consequences of adopting conservation tillage in Europe: Reviewing the evidence. Agric. Ecosyst. Environ..

[112] Mishra A.K., Singh V.P. (2010). A review of drought concepts. J. Hydrol..

[113] Wilhite D.A. (2014). National drought management policy guidelines: A template for action. Integrated Drought Management Programme (IDMP) Tools and Guidelines Series.

[114] Faiz M.A., Zhang Y., Zhang X., Ma N., Aryal S.K., Ha T.T.V., Baig F., Naz F. (2022). A composite drought index developed for detecting large-scale drought characteristics. J. Hydrol..

